# Book Notices

**Published:** 1868-04-01

**Authors:** 


### *BOOK NOTICES.*

---

TREATISE ON THE DISEASES OF THE EYE, Including the Anatomy of the Organ. By Carl Stellwag von Carion, M.D., Professor of Ophthalmology in the Imperial Royal University of Vienna. Translated from the 3rd German edition, by E. C. Hackley, M.D., Surgeon to the New York Eye Infirmary, etc.; and by D. B. St. John Roosa, M.D., Clinical Professor of Diseases of the Eye and Ear in Medical Department of the University of the City of New York, etc. New York: William Wood & Co., 61 Water street. 1868. Pp. 774. For sale by Keen & Co., Lake street, Chicago.

The fact that the three editions of Prof. Stellwag's work in the original have been received with so much praise by ophthalmologists not only in Germany but also in other European countries, together with the fact that for so many years there has been no comprehensive work in English on diseases of the eye, must recommend this translation most emphatically to the profession, wherever our language is spoken.

The general plan of the work, and the manner in which each subject has been discussed, present as little ground for criticism as we could expect in a similar work of any living author.

The reader will find a sufficiently extended statement of all the facts contained in our older authorities, together with the important results given us by so many distinguished observers, who have, more recently, devoted their life labor to the advance of ophthalmic science.

We find each tissue of the eye and its appendages, made the subject of a separate chapter. The minute anatomy, physiology and abnormal changes of each tissue are carefully described, and illustrated, in due order, by numerous cuts. Each disease, its course, results, and treatment, are then fully described.

We can give our readers, perhaps, no better conception of the whole work, than by presenting an abstract of the first section — that devoted to the cornea and its diseases. We first find a description of the anatomy, physiology, senile changes and nosology of the cornea. Then follows a description of the symptoms, causes, course, result and treatment of the several forms of inflammation of the cornea. Pannus, opacities, and staphyloma, as the effects of keratitis, is made each the subject of a sub-section.

There are separate sections, each similar in its general arrangement to the first, devoted to the iris, choroid (glaucoma, sympathetic ophthalmitis), vitreous humor, lens, retina, optic nerve, sclerotic, conjunctiva, lids, lachrymal apparatus, orbit, tumors, artificial eyes, enucleation of the globe, muscles, refraction and "accommodation for vision at different distances," with their anomalies, and the functional (amaurotic) diseases of the optic nerve.

There is an introductory chapter on the treatment of diseases of the eye. Each section closes with a list of authorities upon the subjects under discussion.

In an appendix, we find a short chapter on the ophthalmoscope and its uses, and a series of Jaeger's test types. There is a copious alphabetical index of the whole work.

The classification of diseases is exceedingly simple, and based upon the characteristic appearances shown by direct inspection, or revealed by the ophthalmoscope or *post mortem* (enucleation) examinations. The terminology is also simple; the whole work, from beginning to end, is as free from long Greek compound words, so profusely used by some of the older German ophthalmologists, as such a work at the present time well can be.

A very important feature in the work, is the practical manner in which nearly every subject is treated. There is scarcely any discussion of theories. The author confines himself almost absolutely to the detail of the important and demonstrated facts regarding the anatomy, physiology, pathology and treatment of disease.

The profession is certainly much indebted to Drs. Hackley and Roosa, for this excellent translation of a most valuable work.

The publishers deserve great credit for the perfection of the mechanical execution. The numerous wood engravings and chromo-lithographs add much to the usefulness of the work.

E. L. H.

A Practical Treatise on the Diseases of Women. By T. Gaillard Thomas, M.D., Professor of Obstetrics and Diseases of Women and Children in the College of Physicians and Surgeons, New York; Physician to Bellevue Hospital, etc., etc. With two hundred and nineteen Illustrations. Philadelphia: Henry C. Lea. 1868. Pp. 625.

This book, published in the usual excellent style of the great Philadelphia house, is devoted exclusively to diseases of non-pregnant women — such affections as phlegmasia dolens, puerperal fever, mammitis, etc., not being discussed. The writer has for many years been in a position affording him peculiar advantages for observation; and very considerable experience as a teacher has acquainted him fully with the wants of young practitioners. The work is concise and practical, avoiding the discussion of unsettled questions, but giving a judicious *resumé* of known facts. Not the least interesting feature is its brief historical sketches, which give a bird's-eye view of the progress of the art, from early times.

Atlas of Venereal Diseases. By A. Cullerier, Surgeon to the Hôpital du Midi, Member of the Surgical Society of Paris, Chevalier of the Légion D'Honneur, etc. Translated from the French, with Notes and Additions, by Freeman J. Bumstead, M.D., Professor of Venereal Diseases in the College of Physicians and Surgeons, New York, etc. With about 150 beautifully colored figures, on 26 plates. Philadelphia: Henry C. Lea. 1868. Part I. To be completed in Five Parts. $3.00 each.

A magnificent work — imperial quarto, in the best style of artistic execution. It arrived too late for further notice in the present number.

